# Identification of transient receptor potential melastatin 3 proteotypic peptides employing an efficient membrane protein extraction method for natural killer cells

**DOI:** 10.3389/fphys.2022.947723

**Published:** 2022-09-23

**Authors:** Chandi T. Magawa, Natalie Eaton-Fitch, Cassandra Balinas, Etianne Martini Sasso, Kiran Thapaliya, Leighton Barnden, Rebekah Maksoud, Breanna Weigel, Penny A. Rudd, Lara J. Herrero, Sonya Marshall-Gradisnik

**Affiliations:** ^1^ National Centre for Neuroimmunology and Emerging Diseases, Menzies Health Institute Queensland, Griffith University, Gold Coast Campus, Gold Coast, Qld, Australia; ^2^ Consortium Health International for Myalgic Encephalomyelitis, Griffith University, Gold Coast Campus, Gold Coast, Qld, Australia; ^3^ School of Pharmacy and Medical Sciences, Griffith University, Gold Coast Campus, Gold Coast, Qld, Australia; ^4^ Institute for Glycomics, Griffith University, Gold Coast Campus, Gold Coast, Qld, Australia

**Keywords:** TRP channels, TRPM3, calcium signaling, membrane protein extraction, natural killer cells, TRPM3 proteotypic peptides

## Abstract

**Introduction:** Mutations and misfolding of membrane proteins are associated with various disorders, hence they make suitable targets in proteomic studies. However, extraction of membrane proteins is challenging due to their low abundance, stability, and susceptibility to protease degradation. Given the limitations in existing protocols for membrane protein extraction, the aim of this investigation was to develop a protocol for a high yield of membrane proteins for isolated Natural Killer (NK) cells. This will facilitate genetic analysis of membrane proteins known as transient receptor potential melastatin 3 (TRPM3) ion channels in myalgic encephalomyelitis/chronic fatigue syndrome (ME/CFS) research.

**Methods:** Two protocols, internally identified as Protocol 1 and 2, were adapted and optimized for high yield protein extraction. Protocol 1 utilized ultrasonic and salt precipitation, while Protocol 2 implemented a detergent and chloroform/methanol approach. Protein concentrations were determined by the Pierce Bicinchoninic Acid (BCA) and the Bio-Rad DC (detergent compatible) protein assays according to manufacturer’s recommendation. Using Protocol 2, protein samples were extracted from NK cells of *n* = 6 healthy controls (HC) and *n* = 4 ME/CFS patients. *In silico* tryptic digest and enhanced signature peptide (ESP) predictor were used to predict high-responding TRPM3 tryptic peptides. Trypsin in-gel digestion was performed on protein samples loaded on SDS-PAGE gels (excised at 150–200 kDa). A liquid chromatography-multiple reaction monitoring (LC-MRM) method was optimized and used to evaluate the detectability of TRPM3 *n* = 5 proteotypic peptides in extracted protein samples.

**Results:** The detergent-based protocol protein yield was significantly higher (*p* < 0.05) compared with the ultrasonic-based protocol. The Pierce BCA protein assay showed more reproducibility and compatibility compared to the Bio-Rad DC protein assay. Two high-responding tryptic peptides (GANASAPDQLSLALAWNR and QAILFPNEEPSWK) for TRPM3 were detectable in *n* = 10 extracted protein samples from NK cells isolated from HC and ME/CFS patients.

**Conclusion:** A method was optimized for high yield protein extraction from human NK cells and for the first time TRPM3 proteotypic peptides were detected using LC-MRM. This new method provides for future research to assess membrane protein structural and functional relationships, particularly to facilitate proteomic investigation of TRPM3 ion channel isoforms in NK cells in both health and disease states, such as ME/CFS.

## 1 Introduction

Membrane proteins perform essential and diverse roles in various cellular processes such as catalysis of chemical reactions, movement of ions across membranes, and transmission of signals between cells and their environments (Lin and Guidotti, 2009; Birch et al., 2020). Mutations and misfolding of membrane proteins are associated with various disorders, including neurological indications, the perception of pain, cardiac disorders, kidney failure and blindness (Bagal et al., 2013; Moraes and Quigley, 2021). Given membrane proteins’ diverse tissue distribution and membrane localization, they provide potential targets in proteomic studies, drug discovery and disease management (Lin and Guidotti, 2009; Birch et al., 2020; Moraes and Quigley, 2021). However, membrane protein expression concentrations are low in their native sources. In addition, protein instability, solubility, and contaminations complicate protein extraction attempts, consequently hindering acquirement of membrane proteins of interest from biological samples (Lin and Guidotti, 2009; Kubicek et al., 2014; Birch et al., 2020).

Among the many membrane proteins found in human tissues is the transient receptor potential (TRP) super family of cation channels widely expressed in many tissues (Venkatachalam and Montell, 2007; Nilius and Owsianik, 2010). TRP channels are involved in critical physiological processes, including temperature sensing, taste, vision, nociception, epithelial ion transport, and mineral homeostasis (Venkatachalam and Montell, 2007). Consequently, dysfunction of TRP channels has been associated with several conditions and diseases collectively referred to as TRP-related channelopathies (Nilius et al., 2007; Bréchard et al., 2008; Fraser and Pardo, 2008; Woudenberg-Vrenken et al., 2009; Nilius and Owsianik, 2010). The TRP super family can be subdivided into seven main families on the bases of amino acid sequence homology: TRPC (Canonical), TRPV (Vanilloid), TRPM (Melastatin), TRPP (Polycystin), TRPML (Mucolipin), TRPA (Ankyrin), and TRPN (no mechanoreceptor potential C) (Pedersen et al., 2005; Ramsey et al., 2006; Nilius et al., 2007; Venkatachalam and Montell, 2007). The TRPM subfamily consists of eight different channels (TRPM1-8), including the human transient receptor potential melastatin 3 (hTRPM3) membrane protein which is encoded by the *hTRPM3* gene (Grimm et al., 2003; Lee et al., 2003).

The primary transcript of the hTRPM3 membrane protein is predicted to be 1,555 amino acids long and consists of a TRP signature motif (XWKFXR), six transmembrane domains, an ion transport signature domain between the fifth and sixth transmembrane segments (at amino acids 748-959), and assemble as tetramers forming non-selective cation pores highly permeable for calcium (Ca^2+^) (Grimm et al., 2003; Lee et al., 2003). Hence, hTRPM3 ion channels play a vital role in Ca^2+^ signaling, by mediating Ca^2+^ entry into the cells (Grimm et al., 2003; Lee et al., 2003). Studies have shown that the hTRPM3 gene encodes for over a dozen variants, most of which arise by alternative splicing of the primary transcript (Oberwinkler et al., 2005; Frühwald et al., 2012). Splicing within exon 24 determines the ion selectivity of TRPM3 channels. In addition, alternative splicing of TRPM3 membrane proteins can lead to loss of the protein region indispensable for channel function (ICF), inciting impairment of TRPM3 ion channels (Oberwinkler et al., 2005; Frühwald et al., 2012).

Accumulating evidence has shown TRPM3 ion channel impairment in various neurological conditions and diseases including neurodegenerative disease, glaucoma, intellectual disability, epilepsy, and recently myalgic encephalomyelitis/chronic fatigue syndrome (ME/CFS) (Nguyen et al., 2016; Cabanas et al., 2018; Cabanas et al., 2019; Dyment et al., 2019; Shiels, 2020; Cabanas et al., 2021; Held and Tóth, 2021). ME/CFS is a complex multisystemic disease hallmarked by post-exertional neuroimmune exhaustion and accompanied with symptoms broadly categorized as neurocognitive, autonomic, endocrinological, thermoregulatory, and immunological dysfunctions (Carruthers et al., 2011). Various pathological pathways including altered T cell metabolism, mitochondrial dysfunction, reduced Natural Killer (NK) cell cytotoxicity have been reported in ME/CFS patients (Nguyen et al., 2016; Nguyen et al., 2017; Cabanas et al., 2018; Nguyen et al., 2019; Mandarano et al., 2020; Maksoud et al., 2021). Recent evidence suggests the importance of TRPM3 ion channel dysfunction, and impaired cellular energy metabolism (Carruthers et al., 2011; Christley et al., 2012; Maksoud et al., 2021).

Further investigations are required to determine the role of TRPM3 dysfunction in the pathomechanism of ME/CFS and should aim to investigate TRPM3 proteins in ME/CFS. Currently, membrane protein extraction methods vary widely in viability, reproducibility, and total protein yield due to technical challenges including solubilization and identification (Bünger et al., 2009; Grabski, 2009; Lin and Guidotti, 2009; Janson, 2012; Grant, 2016). In addition, biological sample type, size and intended downstream applications determine the choice of approaches that can be used to process samples, and some techniques are limited to certain cell types and sample sizes (Lin and Guidotti, 2009). Therefore, the primary aim for this current study was to optimize a protein extraction protocol for a high yield of membrane proteins to facilitate the detection of TRPM3 proteotypic peptides for subsequent characterization of isoforms in NK cells isolated from ME/CFS patients and healthy controls (HC).

## 2 Materials and methods

### 2.1 K562 cells preparation and cell culture

The human erthryomyeloblastoid leukemia (K562) cell line was used to optimize protein extraction protocols prior to application in primary NK cells. K562 cells were cultured in Roswell Park Memorial Institute Medium (RPMI)-1640 (Invitrogen Life Technologies, Carlsbad, CA, United States) supplemented with 10% fetal bovine serum (FBS) (Invitrogen Life Technologies, Carlsbad, CA, United States) under physiological conditions in 5% CO_2_ at 37°C. Cultured K562 cells in exponential phase were prepared at 2.0 × 10^6^ and 5.0 × 10^6^ cells per sample in duplicates.

### 2.2 Study participants

ME/CFS patients and HC were sourced from volunteers on the Gold Coast and the National Centre of Neuroimmunology and Emerging Diseases (NCNED) database for ME/CFS. All participants provided written consent and the study was approved by the Griffith University Human Research Ethics Committee (HREC) (ID:2019/1005) and Gold Coast University Hospital HREC (ID:56469). Whole blood samples were collected in ethylenediaminetetraacetic acid (EDTA) tubes between 8:30 a.m. and 10:00 a.m. A total of 23 participants donated 80 ml of whole blood in which NK cells were isolated from prepared peripheral blood mononuclear cells (PBMCs). Of the 23 samples collected, 8 samples were used for the initial optimization stage of protocols, 5 samples for evaluation of protein quantification assay kits and 10 samples for detection of TRPM3 signature peptides.

### 2.3 Peripheral blood mononuclear cell isolation and natural killer cells isolation

PBMCs were isolated from whole blood samples by density gradient centrifugation (Ficoll Paque Plus (Cytiva), GE Healthcare, Uppsala, Sweden). PBMCs were washed and stained with trypan blue stain (Invitrogen) to determine total cell count and cell viability. NK cells were isolated from PBMCs using EasySep human NK cell enrichment kit (Stem Cell Technologies, Vancouver, BC, Canada). On average, approximately 4.0 × 10^6^ NK cells were isolated from each participant. The purity of NK cell isolated was determined by CD3^−^CD56^+^ surface expression using flow cytometry. NK cells were incubated for 20 min at room temperature in the presence of CD3 PE-Cy7 (5µl/test) and CD56 APC (20µl/test) monoclonal antibodies (Becton Dickinson (BD)) Biosciences, San Diego, CA, United States). Cells were acquired at 10,000 events using the Accuri C6 flow cytometer (BD Biosciences, San Diego, CA, United States). Acceptable NK cell purity was ≥90%.

### 2.4 Protein extraction methods

Two protein extraction methods were adapted that utilized lymphoid tissue and whole blood samples reported by (Hanna et al., 2004) and (Scheiter et al., 2013), respectively (Hanna et al., 2004; Scheiter et al., 2013). All procedures and steps were performed on ice or 4°C. Different strategies were applied and evaluated, checking for protein concentration at each step. Protocol 1 utilized an ultrasonic-based technique to disrupt cells and salt precipitation approach combined with ultracentrifugation to isolate and enrich membrane proteins. Protocol 2 utilized a detergent-based technique to disrupt cells and a chloroform/methanol approach to precipitate and isolate membrane proteins. Initially, assessment of the two protocols was undertaken for their protein extraction efficiency of cultured K562 cells. The same protocol adaptations and modifications were applied on primary NK cells isolated from human whole blood samples. Furthermore, assessment of the two protein quantification assays was undertaken: The Pierce BCA and the Bio-Rad DC protein (Lowry) assay kits, to determine their sensitivity and compatibility with the two adapted protocols.

### 2.5 Protein membrane extraction protocol 1

Protocol 1 was adapted from Hanna et al. (2004) that utilized lymphoid tissue samples (Hanna et al., 2004). Membrane protein samples were kept on ice throughout processing to maintain stability and structural integrity. K562 cells and isolated NK cells were washed twice at 4°C with phosphate buffered saline (PBS) 1x solution (Invitrogen Life Technologies, Carlsbad, CA, United States), then once with cold homogenization buffer (0.255 M sucrose, 2 mM EGTA, 2 mM EDTA, 10 mM HEPES, pH7.4). NK cells were lysed in ice cold 100 µl homogenization buffer supplemented with 1x protease inhibitor, applying adjusted ultrasonic vibrations using a Qsonica Ultrasonic Processor sonicator (Amplitude 50%, 30 s, pulse rate 10 s ON/OFF, 5 cycles on ice). The homogenate was centrifuged at 1,500 rpm (Fresco 21 Microliter rotor centrifuge) for 10 min, and the supernatant was collected. The supernatant was subsequently centrifuged at 180,000 × g for 1 h (WX 100 Ultra series Thermo fisher). The pellet was resuspended in salt wash buffer (0.15 M NaCl, 2 mM MgCl2, 20 mM Tris-HCl, pH 7.5), and centrifuged for 180,000 x g for 1 h. Subsequently, the protein pellet was resuspended and boiled at 95–100°C in 1% sodium dodecyl sulfate (SDS) solution, 10 mM Tris, pH 7.5, ready for quantification. The Pierce BCA protein assay (Thermo Fisher Scientific, Waltham, Massachusetts, United States) and Bio-Rad protein assay (Sigma-Aldrich, Roche, Merck KGaA, Darmstadt, Germany) were checked for their compatibility with homogenization buffer, high concentration of salts used for protein precipitation and detergent used for protein solubilization.

### 2.6 Protein membrane extraction protocol 2

Protocol 2 was adapted from Scheiter et al. (2013) that utilized whole blood samples from human donors. All procedures were performed on ice to maintain membrane protein stability and structural integrity. K562 cells and NK cells were lysed in 100 µl cold lysis buffer (50 mM HEPES pH 7.5, 150 mM NaCl, 1% Triton-X100, 1 × protease inhibitor mixture supplemented with EDTA) for 30 min. Lysates were centrifuged at 16,000 x g for 20 min. Supernatants were collected and methanol/chloroform precipitation was used to precipitate membrane proteins as previously described (Janson, 2012). Protein pellets were resuspended in 50 µl 1% SDS and quantified using a Nanodrop spectrophotometer (ND-1000). Compatibility check was performed for the Pierce BCA and Bio-Rad protein assays with the lysis buffer and protein solubilization detergent used.

### 2.7 Quantification of protein yield

For total protein yield detection, the Pierce BCA Protein Assay kit with bovine serum albumin (BSA) protein as a reference standard and the Bio-Rad DC Protein (Lowry) assay kit with bovine gamma globulin (BGG) protein as a reference standard were simultaneously used for both protocols (using 10 and 5 µl of protein samples for BCA and Lowry respectively). Protein quantifications were performed following manufacturer instructions and biological samples were prepared under the same conditions as standards. The two standard curves were plotted following manufacturers’ recommended procedures, and the two protein quantification assays were evaluated and compared for their sensitivity and compatibility with the composition of buffers and protein solubilization detergents used. Absorbance values were plotted against known protein standard concentrations to determine the equation of the straight line and regression coefficient (R2). Extracted protein sample concentrations were calculated and estimated from plotted standard curve equations.

### 2.8 Preparation of samples for LC-MRM analysis by SDS-PAGE gel analysis

Sodium dodecyl sulfate-polyacrylamide gel electrophoresis (SDS-PAGE gel) analysis was performed to separate and detect TRPM3 proteins from heterogenous mixtures of protein samples. Laemmli buffer 5 × (0.05% Bromophenol blue, 300 mM Tris-HCl, 5% SDS, 50% Glycerol, and 250 mM Dithiothreitol (DTT)] was added to extracted protein samples to a 1 X dilution (1 mg/ml protein concentration). Thirty Five Microliters of 1 mg/ml (40 µg) of protein samples were loaded on SDS-PAGE gels (4%–12% NuPAGE Bis Tris SDS-PAGE gels). After migration, bands of gel were excised with a scalpel between 150 and 200 kDa (estimated TRPM3 monomer molecular weight range). Prepared gel bands were first de-stained with successive washes of 25 mM ammonium bicarbonate, 25 mM acetonitrile and 25 mM bicarbonate, followed by a final wash with 100% acetonitrile. Each band was reduced with 200 µl of 10 mM Tris (2-carboxyethyl) phosphine (TCEP), incubated at 56°C, for 30 min, alkylated with 200 µl of 55 mM iodoacetamide for 30 min in the dark, then washed/incubated in 100% acetonitrile for 15 min. Each gel band was digested overnight at 37°C, in 50 µl of trypsin digest solution (20 µg trypsin resolubilized with 600 µl of 25 mM bicarbonate). To extract peptide digests, 70 µl of 25 mM ammonium bicarbonate with 50% acetonitrile was added to each sample, incubated/centrifuged at 800 rpm at room temperature for 15 min, and the supernatant was transferred to a low-binding tube. 70 µl of 25 mM ammonium bicarbonate with 5% formic acid was added to each tube and incubated/centrifuged at 800 rpm at room temperature for 15 min. The supernatant was transferred to new low-binding tubes. 70 µl of 100% acetonitrile was added to each tube, incubated/centrifuged at room temperature for 15 min, supernatant was transferred to a new low-binding tube and dried in a speed vacuum.

### 2.9 Targeted protein analysis by liquid chromatography-multiple reaction monitoring

**FIGURE 1:**
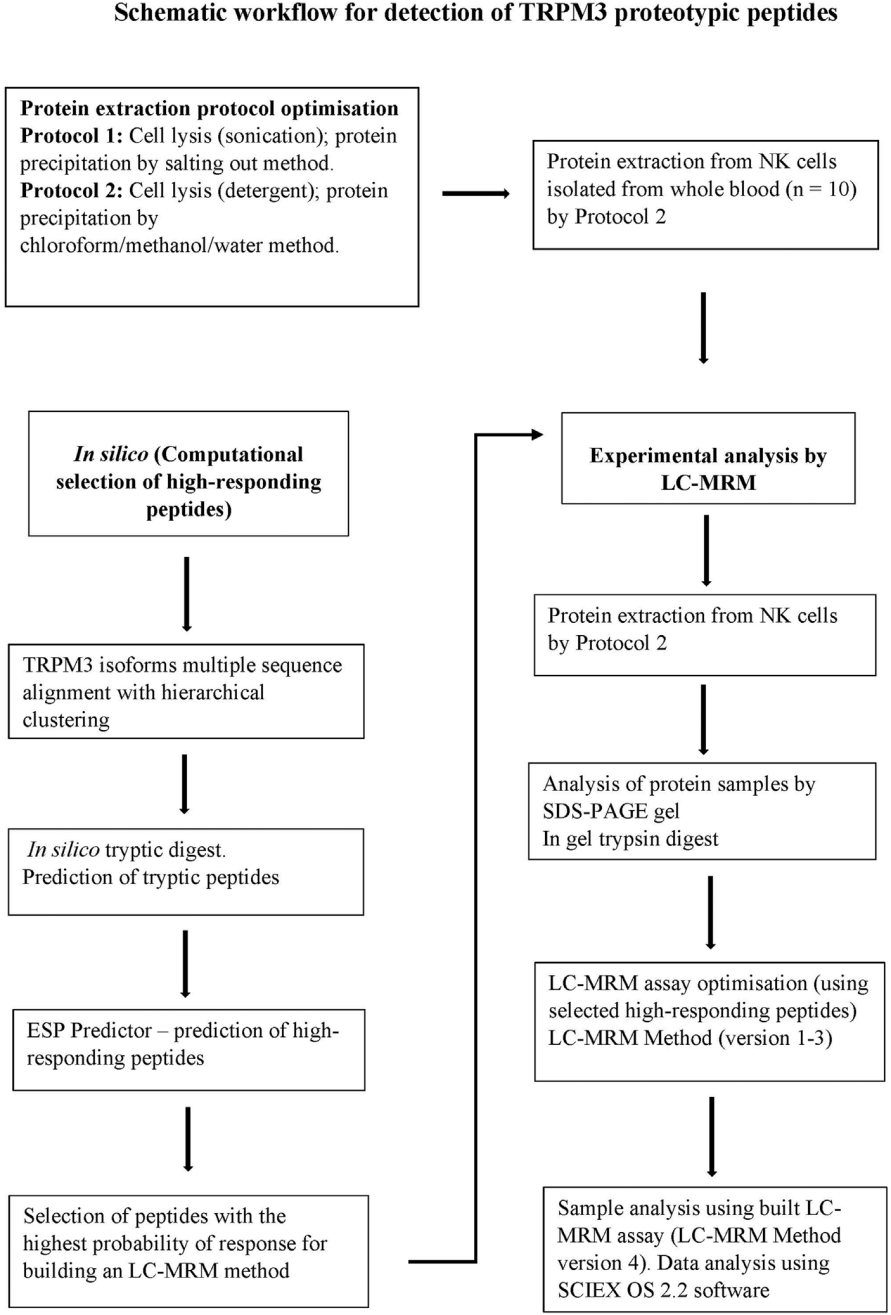
A schematic workflow for the detection of TRPM3 signature peptides by LC-MRM method. TRPM3 isoform sequences were computationally digested *in silico* to produce a set of predicted tryptic peptides. Tryptic peptides were input into the enhanced ESP predictor and a subset of high-responding peptides (peptides with the highest ion-current response deemed more likely to provide the best detection response) were selected to build the experimental LC-MRM method. LC-MRM method versions 1–3 were employed during optimization monitoring peptides within a selected time window (increasing signal intensity and improving detection of peaks). Undetectable peptides were removed from the final method. LC-MRM Method version 4 was finally employed to target and monitor a few selected high-responding TRPM3 surrogate peptides in extracted protein samples, evaluating detectability of the monitored peptides.

To monitor and evaluate detectability of TRPM3 peptide sequences in extracted protein samples, liquid chromatography-multiple reaction monitoring (LC-MRM) assay was performed by Promise Proteomics, Grenoble, France. A schematic workflow illustrating procedures performed is shown in Figure 1. Initially, amino acid sequences for human TRPM3 isoforms (a-f) were retrieved from the National Center for Biotechnology Information (NCBI) and the Universal Protein Resource (UniProt) databases. Computational hTRPM3 isoform sequence alignment was performed using multiple sequence alignment with hierarchical clustering method (Scheiter et al., 2013). *In silico* tryptic digest was first performed to select tryptic peptides for TRPM3 isoforms. Enhanced signature peptide (ESP) predictor was used to predict high responding TRPM3 signature peptides. Peptides with the highest probability of response and highest rates of being detected by liquid chromatography coupled with mass spectrometry (LC-MS) were ranked and selected. The best high-responding peptides were used to optimize and build an experimental LC-MRM method. Experimental analysis of protein samples was then performed using the built LC-MRM assay. Internal quality controls (QC) for the mass spectrometer instrument were used at the beginning and end of the batch. The LC-MRM analyses were performed on a chromatographic module Exion LCTM (SCIEX), connected to a mass spectrometer instrument QTRAP^®^ 6500+ (SCIEX). Peptides were applied to a precolumn, Acclaim PepMap C18 5 µm 1.0 × 15 mm 100 A (Thermo Fisher Scientific -160438), Chromatographic column: Biozen Peptide XB-C18 2.6 µm, 100 × 2.1 (Phenoemenex-00D-4768-AN), Total run time: 10 min.

### 2.10 Biological and chemical reagents

Ficoll paque plus used to isolate PMBCs was purchased from Bio-Strategy (product code: GEHE17-1440-03). EasySep human NK cell enrichment kits (product code: 19055) and EasySep buffer (product code: 20144) were purchased from Stem Cell Technologies (product code: 19055). The Complete TM EDTA-free protease inhibitor cocktail was purchased from Sigma-Aldrich (product code: 11873580001) and stored according to manufacturer’s recommendations. RPMI 1640 (product code: 11835030) used for the culture of K562 cells and PBS (product code: 20012050) were purchased from Life Technologies. The Pierce BCA protein assay kit (product code: 23225) was purchased from ThermoFisher Scientific, while the Bio-Rad DC protein assay kit (product code: 6454) was purchased from Bio-Rad Laboratories. The following reagents were purchased from Sigma Aldrich: Molecular biology Triton TM X-100 (product code: T8787); Sucrose (product code: S0389); DTT (product code: 10708984001); Glycerol (product code: G5516); bromophenol blue (product code: 1610404); Tris-HCL (product code: T3038); SDS (product code: 05030); Chloroform (product code: 288306); and Methanol (product code: 322415). HEPES solution (product code: 15630080) was purchased from ThermoFisher Scientific.

### 2.11 Statistical analysis

Data were analyzed using GraphPad Prism, version 9 (GraphPad software Inc., version 9, CA, United States). Normality was determined using the Shapiro Wilk test. To determine statistical significance between groups in total protein yield, the independent *t*-test was performed. Significance was set at *p* < 0.05 and data is presented as mean ± standard error of the mean (SEM).

## 3 Results

### 3.1 Participant demographics

A total of 23 participants were included for this present study, *n* = 15 HC and *n* = 8 ME/CFS patients. *N* = 13 participants were used for evaluation of protein concentration in samples and *n* = 10 participants were used for detection of TRPM3 signature peptides by LC- MRM assay. The mean ages of HC and ME/CFS patients were 40.47 ± 16.25 and 45 ± 0.0, respectively. For HC, *n* = 4 were males and *n* = 11 were females, and for ME/CFS patients *n* = 2 were males and *n* = 6 were females. Table 1 outlines relevant demographic information of participants.

**TABLE 1:** Demographic results of participants.

Parameters	HC(*n* = 15)	ME/CFS (*n* = 8)	*p* value
	Protein concentration evaluation (*n* = 9)	Protein concentration evaluation (*n* = 4)	
	Peptide detection (*n* = 6)	Peptide detection (*n* = 4)	
Age (years) (Mean ± SD)	40.47 ± 16.25	45 ± 0.0	0.5388
Gender			
Male (n%)	4 (26.7%)	2 (25.0%)	
Female (n%)	11 (73.3%)	6 (75.0%)	

Abbreviations: HC, healthy control; ME, myalgic encephalomyelitis; CFS, chronic fatigue syndrome; SD, standard deviation.

### 3.2 Comparison of total membrane protein yield by the two protein extraction methods

**FIGURE 2:**
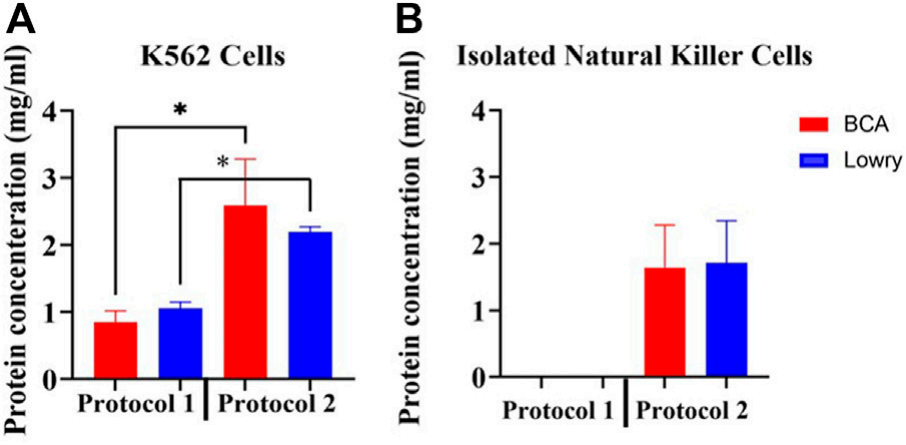
Determination of protein extraction efficiency by the two adapted protocols. **(A)** Comparison of average total membrane protein yield from cultured K562 cells using the two adapted protocols. 2 × 10^6^ K562 cells were used per sample. For both protocols, protein quantification was performed using the Pierce BCA and Bio-Rad DC protein assays simultaneously using a NanoDrop spectrophotometer. Normality was determined using the Shapiro Wilk test (*p* > 0.05). Data is represented as the mean ± SEM as determined by independent *t*-test. *p < 0.05. **(B)** Average total protein yield from NK cells isolated from 5 human donor whole blood using Protocol 2. The Pierce BCA and Bio-Rad DC protein assays were simultaneously used to detect protein concentrations in each sample. Abbreviations: Bio-Rad DC protein assay (Lowry), detergent-based protein extraction method (Protocol 2), myelogenous leukemia cell line (K562 Cells), Pierce Bicinchoninic Acid protein assay (BCA), ultrasonic-based protein extraction method (Protocol 1).

Total membrane protein yield was significantly different (*p* < 0.05) between the two protocols (Figure 2A). Protocol 2 consistently yielded significantly higher total protein from cultured K562 cells than Protocol 1, as measured by both the Pierce BCA (2.5876 mg/ml vs. 0.8450 mg/ml, *p* = 0.0028) and the Bio-Rad DC (2.1940 mg/ml vs. 1.0595 mg/ml, *p* = 0.0001) protein assay kits.

An average of 4.0 × 10^6^ NK cells per sample were used for extraction of membrane proteins from isolated NK cells using Protocol 2. Average NK cell purity was ≥90% (Gating strategy illustrated in Supplementary Material 1.0). Average protein yield from NK cells was 1.641 mg/ml with the Pierce BCA protein assay and 1.713 mg/ml with the Bio-Rad protein assay (Figure 2B). There was no significant difference (*p* = 0.0756 and *p* = 0.3033 in Protocol 1 and Protocol 2, respectively) in protein concentration detection by the two protein quantification assays per sample. No protein concentrations could be detected in samples when using Protocol 1 to extract membrane proteins from NK cells.

### 3.3 Evaluation of compatibility of the two protein assay kits with protocol 2 lysis buffer and protein solubilization detergent

**FIGURE 3:**
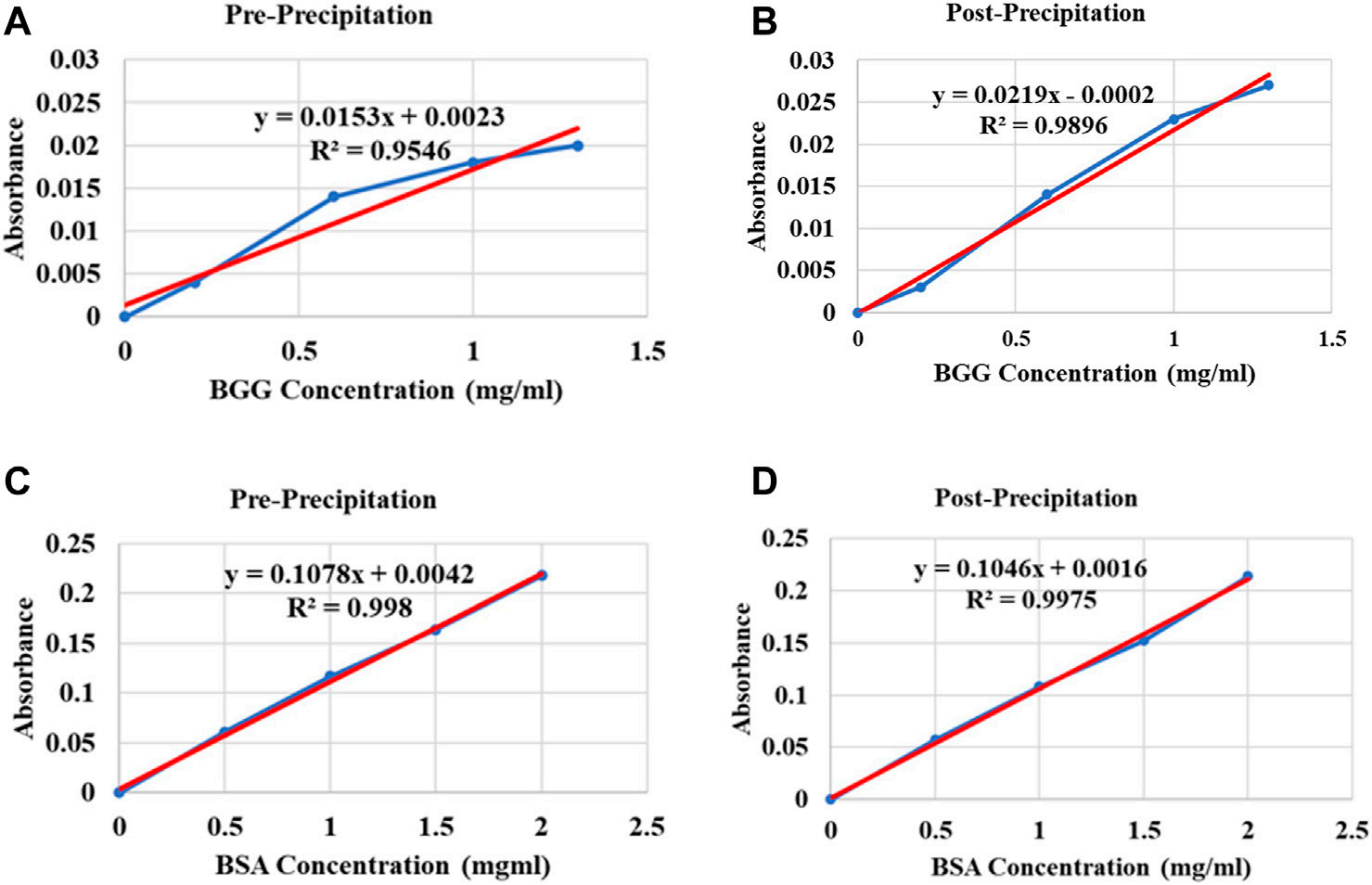
Standard curve plot examples of the Bio-Rad DC (Lowry) protein assay and the Pierce BCA protein assay illustrating variations between the two protein assay standard curve plots using Protocol 2 buffers. **(A)** Compatibility illustration of the Bio-Rad DC protein assay kit with Protocol 2 lysis buffer: pre-precipitation, **(B)** protein solubilization detergent: post-precipitation. Different concentrations of BGG protein as reference protein standard diluted in lysis buffer and solubilization detergent respectively, were plotted against absorbance values. **(C)** Compatibility illustration of the Pierce BCA protein assay kit with Protocol 2 lysis buffer: pre-precipitation, **(D)** protein solubilization detergent: post-precipitation. Different concentrations of BSA protein as reference protein standard diluted in lysis buffer and solubilization detergent respectively, were plotted against absorbance values. Abbreviations: Bovine serum albumin (BSA), Bovine gamma globulin (BGG).

There was no significant difference in protein concentration detection by the two protein quantification assays; however, the Bio-Rad DC (Lowry) protein assay linear response curves were difficult to reproduce and less consistent compared to the Pierce BCA protein assay linear response curves. As a result, the Bio-Rad DC protein assay appeared to overestimate protein concentrations in samples. For the Bio-Rad DC assay, pre-precipitation and post-precipitation linear response curve adjusted R_2_ = 0.9291 and 0.9861 respectively, and for the Pierce BCA assay, pre-precipitation and post-precipitation linear response curve adjusted R_2_ = 0.9973 and 0.9967, respectively. Results are shown in Figure 3.

### 3.4 Detection of TRPM3 proteotypic peptides by liquid chromatography-multiple reaction monitoring method

#### 3.4.1 Preparative purification of protein samples by Sodium dodecyl sulfate-polyacrylamide gel electrophoresis

**FIGURE 4:**
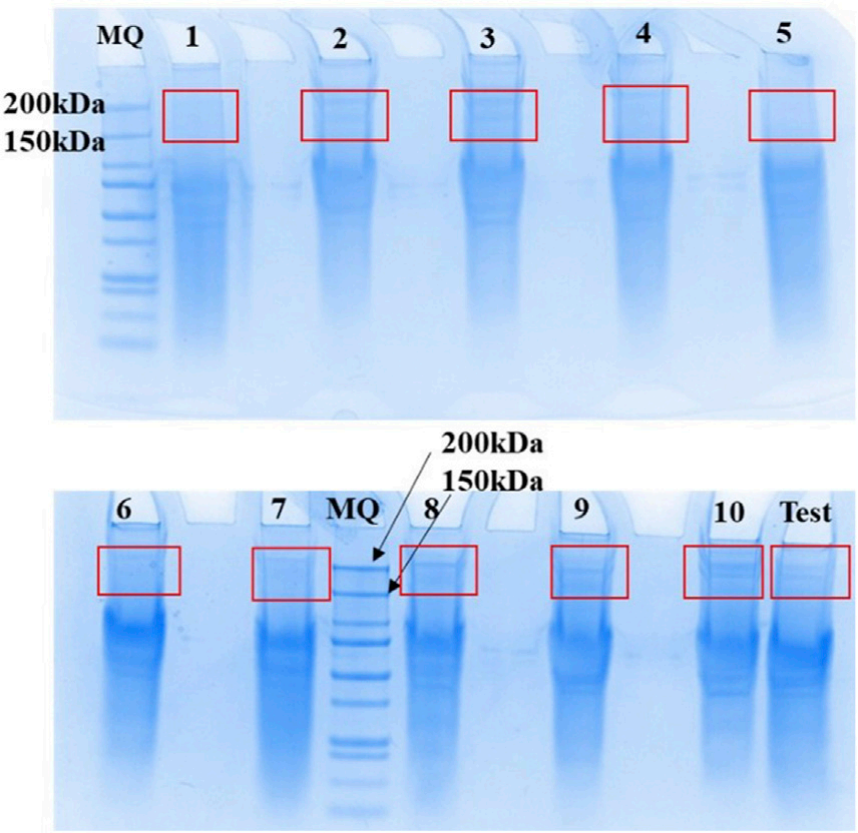
Elution and recovery of TRPM3 membrane proteins separated by SDS-PAGE. Membrane proteins extracted from n = 10 human whole blood using the adapted Protocol 2 were purified using SDS-PAGE PAGE gels (4–12% NuPAGE Bis Tris SDS PAGE gels) at a concentration of 1 mg/ml. A prepared TRPM3 internal standard (MQ) was used, and after migration bands of gel between 150 kDa and 200 kaDa (expected TRPM3 MW) were excised with a scalpel as illustrated in Figure, for in-gel digest of protein samples. Figure obtained from Promise Proteomics.

SDS-PAGE gel was used to purify membrane protein samples extracted from whole blood of *n* = 10 participants using Protocol 2 (Results illustrated in Figure 4). TRPM3 membrane proteins were localized on the gel and identified between 150 and 200 kDa bands, which correspond to the expected molecular weight of TRPM3 monomer in all 10 samples.

#### 3.4.2 In silico tryptic digest and prediction of high-responding TRPM3 tryptic by the enhanced signature peptide predictor

Tryptic peptides were selected following *in silico* tryptic digest of TRPM3 canonical sequences. The ESP predictor was used to predict high responding TRPM3 tryptic peptides and 15 high responding TRPM3 tryptic peptides with unique amino acid sequence and the highest probability of response (with scores close to 1 ranked as best) were selected for building an LC-MRM method. Specific analyte molecular ions and several of their fragment ions were selectively monitored. Suitable MRM transitions (predefined mass-to-charge ratio corresponding to a precursor peptide ion and several specific fragment peptide ions) of the target peptide were selected. Peptides with the best ionization transitions with a potential to provide the best intensity and specificity were selected. Following optimization of the LC-MRM assay, five high responding peptides with the best ionization transitions were selected as quantitative surrogates for TRPM3 protein, results are shown in Table 2.

**TABLE 2:** Identification of high responding tryptic peptides.

Peptide	Precursor (*m/z*)	Fragment ion (*m/z*)	Retention time (minutes)	Collision energy
GANASAPDQLSLALAWNR	927.98+	1043.60+	6.45	42.3
		930.52+		
		843.48+		
		730.40+		
		659.36+		
		546.28+		
NWSNATCLQLAVAAK	823.92+	1145.63+	5.01	38.5
		1074.60+		
		813.52+		
		700.44+		
		572.38+		
		673.86++		
EILMSEPGK	502.26+	874.47+	4.87	26.9
		761.39+		
		648.30+		
		517.26+		
QAILFPNEEPSWK	779.90+	1133.52+	4.12	36.9
		986.46+		
		889.41+		
		775.36+		
		646.32+		
		680.35++		
		623.81++		
SIDFEDITSMDTR	765.34+	1067.47+	6.32	36.4
		938.42+		
		823.40+		
		710.31+		
		609.27+		
		522.23+		
		721.83+		

Abbreviations: m/z, mass-to-charge ratio.

#### 3.4.3 Detection of TRPM3 signature peptides by targeted liquid chromatography-multiple reaction monitoring assay

**FIGURE 5:**
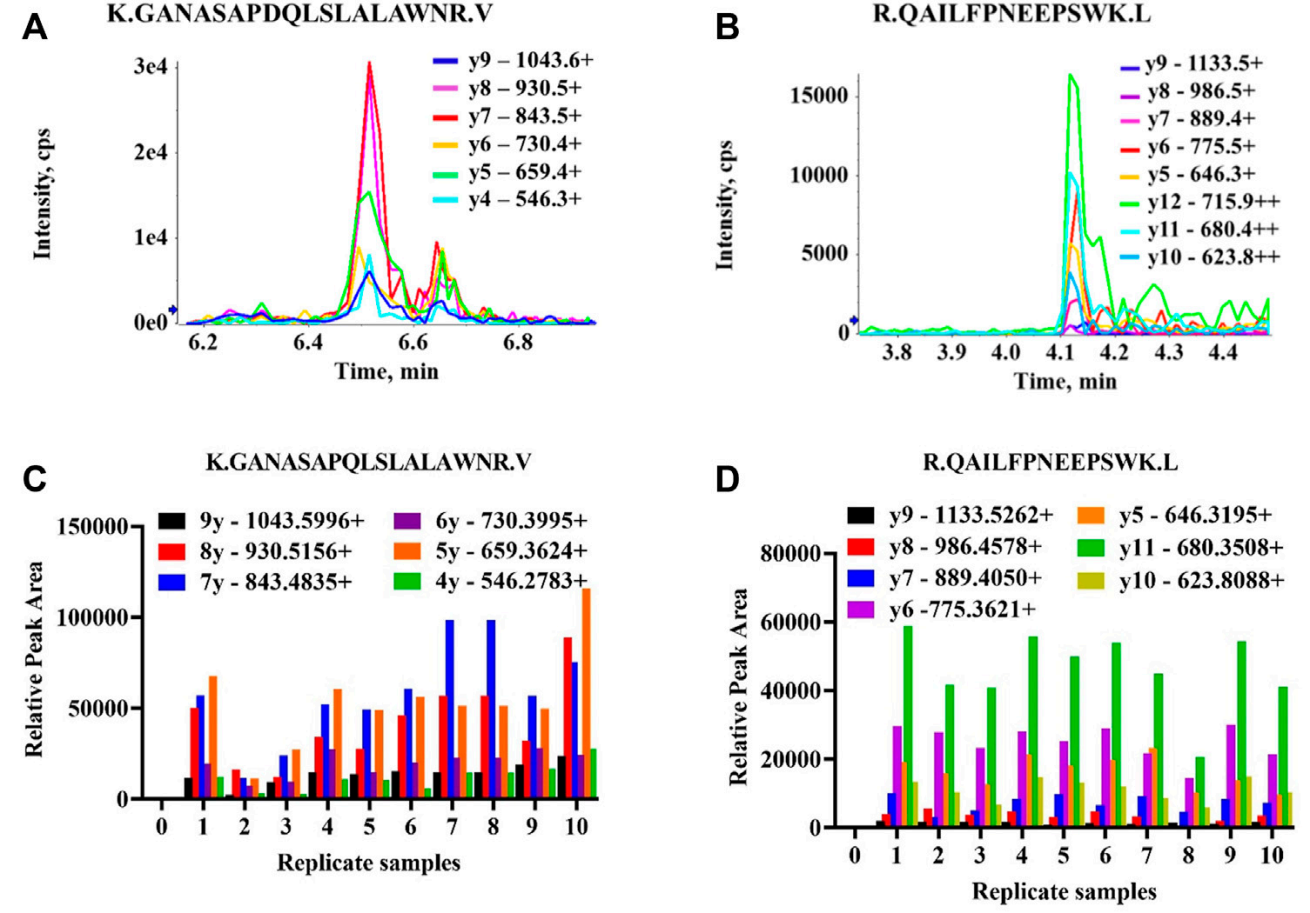
Detectability of TRPM3 proteotypic peptide sequences. All transitions (precursor/fragment ion pairs) per peptide were monitored over time yielding a set of chromatographic traces with the retention time and relative signal intensities as coordinates. The resulting MRM peaks were then evaluated for their capability to specifically detect target peptides. **(A)** MRM traces of six transitions for K.GANASAPDQLSLALAWNR.V peptide. One peak with multiple MRM transitions (parent ion/daughter ions) co-eluting at 6.45 min in one participant protein sample. Each series represents product y ions (ionized amino acids/fragmented peptides) which collectively form target peptide sequences. **(B)** MRM traces of eight transitions for R.QAILFPNEEPSWK.L peptide. One peak with multiple MRM transitions co-eluting at 4.12 min **(C,D)** Relative peak areas for multiple MRM transitions co-eluting at the same retention time (6.45 and 4.12 min) detectable across all ten replicate samples (Supplementary Material) for K.GANASAPDQLSLALAWNR.V and R.QAILFPNEEPSWK.L peptides, respectively.

Preliminary results indicated two TRPM3 proteotypic peptides were detectable in all 10 human protein samples extracted from ME/CFS patients and HC. Results are illustrated in Figure 5. GANASAPDQLSLALAWNR and QAILFPNEEPSWK peptides had multiple MRM transitions detected at the same retention time in all 10 protein samples. For GANASAPDQLSLALAWNR, co-eluting transitions were apparent at 6.45 min and for QAILFPNEEPSWK at 4.12 min. SIDFEDITSMDTR peptide showed multiple MRM transitions detection at 6.3 min in 3 protein samples only (Supplementary Material 1.1). Limited MRM transitions were seen for NWSNATCLQLAVAAK and EILMSEPGK peptides in all 10 protein samples.

## 4 Discussion

This preliminary investigation aims to evaluate the detectability of TRPM3 proteotypic peptides by LC-MRM method following extraction of protein samples from NK cells isolated from HC and ME/CFS patients using two adapted protein extraction protocols. For the first time, we were able to detect two TRPM3 proteotypic peptides by LC-MRM method in extracted human protein samples. Multiple MRM transitions were detectable at the same retention time for each of the two detected peptides across all ten samples. Furthermore, results from this study concur with previous investigations which reported variations in viability, reproducibility, and final protein yield by existing membrane protein extraction protocols. Therefore, our primary objective was to adapt and optimize a method to potentially enable the structural and functional proteomic analyses of membrane proteins.

Membrane proteins extracted from biological samples cannot be amplified for analytical purposes (Bréchard et al., 2008; Fraser and Pardo, 2008). Therefore, a high yield for membrane proteins from biological samples relies on optimization of protein extraction strategies. There are critical factors and parameters to consider when selecting the most viable and efficient method for collecting and analyzing membrane proteins (Janson, 2012). Membrane protein isolation is a multiple steps process, and various approaches are used to disrupt cell membranes including mechanical-based techniques such as sonication, French press and grinding as well as nonmechanical-based approaches such as osmotic shock, freeze thawing and detergent-based approaches (Lin and Guidotti, 2009; Janson, 2012). Cell lysis liberates various cell contaminating molecular species, inevitably disrupting, and potentially compromising the structural and functional integrity of membrane proteins (Birch et al., 2020; Moraes and Quigley, 2021). Removal of interfering substances can be achieved by various techniques including acid, salt, and alcohol precipitation methods, usually combined with differential centrifugation (Janson, 2012; Birch et al., 2020; Moraes and Quigley, 2021). Therefore, to evaluate the best approach for lysing NK cells and precipitate membrane proteins, a comparison of two methods which employed different protein extraction strategies was performed.

This study found significant difference in final protein yields between the two protocols, with the protein yield from K562 higher when utilizing Protocol 2, compared with protein concentration yield when using Protocol 1. This current adapted protocol is supported by previous investigations that reported wide variations in protein yield and reproducibility by existing protein extraction methods, which has been widely reported as a big challenge in membrane proteomic studies (Alberts et al., 2002; Bünger et al., 2009; Grabski, 2009; Lin and Guidotti, 2009; Janson, 2012; Lai, 2013; Scheiter et al., 2013; Kubicek et al., 2014; Grant, 2016; Lee, 2017; Reis and Moraes, 2019; Birch et al., 2020; Moraes and Quigley, 2021). No one cell disruption approach is better since each of these approaches has limitations and challenges. For example, ultrasonic-based approach is more suitable for batch processing; however, there is risk of producing moderate to fine protein particles which are unsuitable for some downstream applications (Lai, 2013). In contrast, other cell lysis approaches such as osmotic shock are very gentle; however, such techniques may be insufficient for other cell types (Lai, 2013).

Additionally, both methods were evaluated for their suitability to extract viable membrane proteins from NK cells. Unfortunately, no proteins could be detected in samples at the final stages of Protocol 1 by both the Pierce BCA, and the Bio-Rad DC (Lowry) assay kits, hence Protocol 2 was deemed viable for assessment of membrane proteins. For Protocol 1, high energy used to disrupt cell membranes by sonication may have been incompatible for NK cells isolated from blood samples. The procedure involved fragmenting membrane proteins into exceedingly small particles that were difficult to pellet at higher forces (180,000 ×g) resulting in low final protein yield. Alternatively, when samples were centrifuged at a higher acceleration of 240,000 ×g, protein pellets formed were exceedingly difficult to resuspend or solubilize. Therefore, Protocol 2 was concluded as the most suitable and viable protein extraction method for NK cells isolated from whole blood in conjunction with additional modifications for our study. Although the detergent-based method was more efficient for extraction of membrane proteins in this study, many commercial grade detergents are associated with increased levels of carbonyl compounds, peroxides and sulfhydryl oxidizing agents which interfere with protein structure and peptide detection. This current project employed molecular biology or proteomic grade Triton X-100 which contain reduced peroxides and carbonyl compounds making it suitable for protein purification and quantitation (Pham et al., 2016).

Furthermore, the two protein quantification assay kits were evaluated for their sensitivity and compatibility with Protocol 2. Protein quantification methods such as the Bradford (Coomassie G-250) protein assay, the Pierce BCA assay, and the Bio-Rad DC (Lowry) assay are commonly utilized for total protein detection (Noble and Bailey, 2009; Goldring, 2012; Hussain et al., 2019). However, selection of protein quantification assays is influenced by their compatibility with the composition of precipitation buffers and solubilization detergents used, quantity of protein in samples, detection limit of method and preparation time (Noble and Bailey, 2009; Goldring, 2012). For this study, the Pierce BCA and the Bio-Rad DC assays were simultaneously utilized for detection of protein concentration in samples. Both methods efficiently quantified protein concentrations; however, there were variations in the standard curve plots produced by the two protein assay kits. In general, the Bio-Rad DC protein assay standard curve was difficult to reproduce, and protein concentration detection varied between samples indicating sample interference intolerance, resulting in potential overestimation of protein concentrations in samples. Nevertheless, an argument in favor of the Bio-Rad DC protein assay is that it showed more sensitivity as it requires less protein sample volume per assay and less preparation time compared with the Pierce BCA protein assay. Composition of protein extraction buffers and solubilization detergents is known to interfere with some protein quantification assays, hence the need to check compatibility of reagents (Goldring, 2012; Janson, 2012; Grant, 2016).

Finally, the detectability of TRPM3 peptide sequences in extracted protein samples was evaluated by LC-MRM method. In this preliminary investigation, two TRPM3 peptides GANASAPDQLSLALAWNR and QAILFPNEEPSWK had multiple MRM transitions detected at the same retention time in all 10 samples. For the SIDFEDITSMDTR peptide (Supplementary Material), multiple MRM transitions could only be detected in three samples, and this peptide sequence is missing in some hTRPM3 isoforms. Therefore, future studies should further investigate if the SIDFEDITSMDTR peptide could be used to distinguish or identify different TRPM3 isoforms and further investigate their structural and functional characteristics in extracted protein samples.

LC-MS is a powerful technique that can be used to detect and quantify peptides sequences in protein samples (Fusaro et al., 2009; Picotti and Aebersold, 2012; Tang et al., 2022). In the LC-MRM method, analytes in ionized form are detected by the mass spectrometer, following fragmentation of selected precursor ions by collision-induced dissociation (CID) and the method minimizes interferences from other particles (Picotti and Aebersold, 2012; Tsai et al., 2016). Proteotypic peptides can be selected as a stoichiometric representative of protein from which it was cleaved; however, this does not fully correlate to signal intensity (Picotti and Aebersold, 2012; Tsai et al., 2016; Tang et al., 2022). Although five TRPM3 peptides were identified as having the best ionization transitions *in silico*, only two TRPM3 proteotypic peptides could be detected in all samples. Therefore, the optimized protein extraction protocol managed to produce membrane proteins that were shown to provide reliable identification of TRPM3 peptides in heterogenous mixtures of protein samples by LC-MRM assay.

One of the present limitations with our preliminary results is the absence of protein extraction data for NK cells utilizing Protocol 1. While a comparison of the quality of peptides produced by the two different techniques would have been insightful, this was not undertaken due to the success of Protocol 2 in K562 cells. Another limitation is the absence of tandem mass spectrometry (MS/MS) data to validate and quantify detected peptides. In addition, the current investigation focused on detectability of TRPM3 peptides in extracted protein samples for protocol optimization purposes only, without comparing negative and positive controls. Future analysis to confirm these preliminary results should be confirmed by MS/MS sequencing using high resolution mass spectrometry (HRMS) employing negative and positive controls. Furthermore, future investigations should confirm the identification and differentiation of peptides from different TRPM3 isoforms. However, the absence of complete sequencing of target protein can limit the proteomic investigations, meaning complete target peptides database are essential for peptide sequences to be identifiable by proteomic tools, ensuring quality assurance. Additionally, absence of fully characterized proteome equivalent to fully sequenced genome increases challenges in proteomic studies, as the number of potential modifications to a protein structure that can change its functions are numerous (Lange et al., 2008; Picotti and Aebersold, 2012).

In conclusion, isolation of membrane proteins from primary NK cells was successful using the detergent-based protein extraction protocol. Furthermore, the Pierce BCA protein assay was more reproducible and compatible with the optimized protocol. Based on LC-MRM preliminary data, the adapted protocol managed to produce detectable TRPM3 proteotypic peptides in heterogenous mixtures of extracted protein samples. Optimization of this protocol now informs future functional and structural proteomic research and extends our understanding of TRPM3 ion channels in NK cells of ME/CFS patients. Using this method, the analysis of TRPM3 ion channels in NK cells of ME/CFS patients may gain additional insight into the pathomechanism of this illness and assess potential diagnostic/screening tests.

## Data Availability

The original contributions presented in the study are included in the article/Supplementary Material, further inquiries can be directed to the corresponding author.

## Glossary

BC: Becton Dickinson
BCA: Bicinchoninic acid
BGG: Bovine gamma globulin
BSA: Bovine serum albumin
CID: Collision induced dissociation
DC: Detergent compatible
DTT: Dithiothreitol
EDTA: Ethylenediaminetetraacetic acid
ESP: Enhanced signature peptide
FBS: Fetal bovine serum
HC: Health controls
HREC: Human Research Ethics Committee
HRMS: High resolution mass spectrometry
hTRPM3a: Human transient receptor potential melastatin 3 (a)
ICF: Indispensable for channel function
K562: Human erythromyeloblastoid leukemia cell line
LC-MRM: Liquid chromatography-multiple reaction monitoring
LC-MS: Liquid chromatography mass spectrometry
ME/CFS: Myalgic encephalomyelitis/chronic fatigue syndrome
mTRPM3α2: Mouse transient receptor potential melastatin 3 alpha 2
MS/MS: Tandem mass spectrometry
NCBI: National Center for Biotechnology Information
NCNED: National Centre for Neuroimmunology and Emerging Diseases
NK: Natural Killer cells
PBMC: Peripheral blood mononuclear cell
PBS: Phosphate buffered saline
QC: Quality Control
R2: Regression coefficient
RPMI: Roswell Park Memorial Institute
SDS: Sodium dodecyl sulfate
SDS-PAGE: Sodium dodecyl sulfate polyacrylamide gel electrophoresis
SEM: Standard error of the mean
TCEP: Tris(2-carboxyethyl) phosphine
TRP: Transient receptor potential
TRPA: Transient receptor potential ankyrin
TRPC: Transient receptor potential canonical
TRPM3: Transient receptor potential melastatin 3
TRPML: Transient receptor potential mucolipin
TRPN: Transient receptor potential no mechanoreceptor potential C
TRPP: Transient receptor potential polycystin
TRPV: Transient receptor potential vanilloid
UniProt: Universal Protein Resource
