# Supplementary material for: Identification of transient receptor potential melastatin 3 proteotypic peptides employing an efficient membrane protein extraction method for natural killer cells
Source: Front Physiol. 2022 Sep 23;13:947723. doi: 10.3389/fphys.2022.947723 (PMC9540229; doi:10.3389/fphys.2022.947723)
Supplement: Supplementary file 1 [file Table1.DOCX]

## Supplementary material


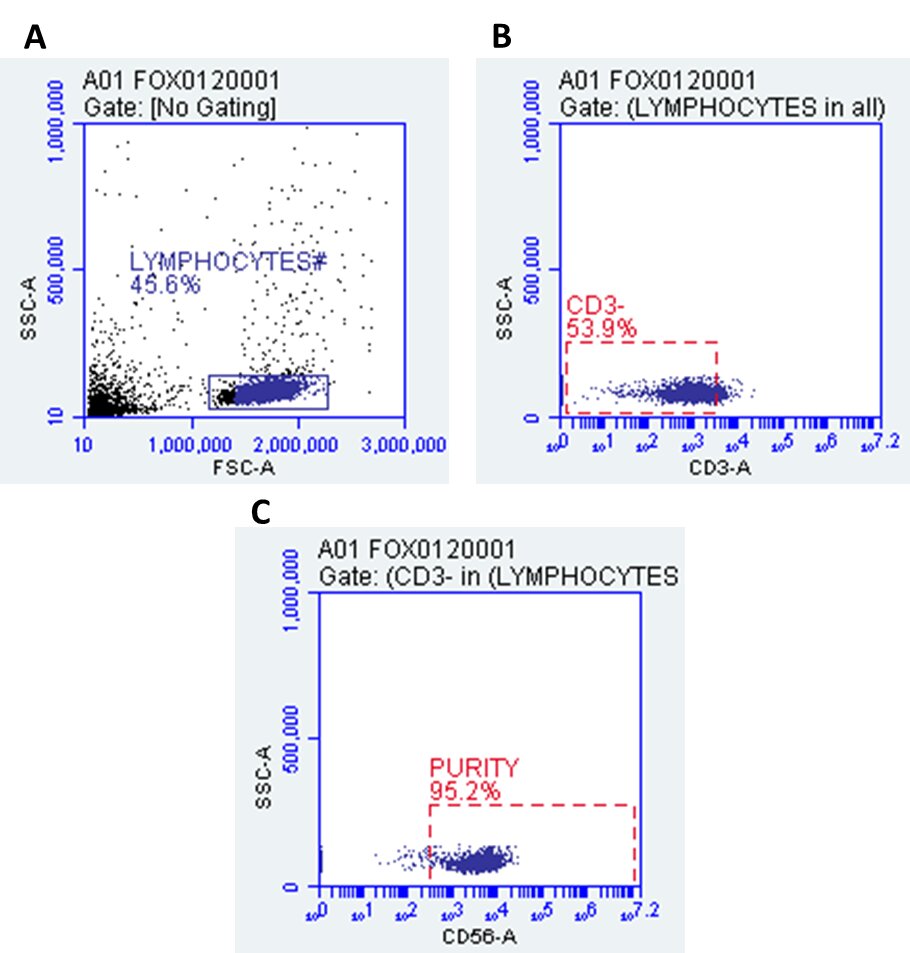


**Figure 1:** Supplementary material 1.0. Flow cytometry gating strategy used for NK cell purity. (A) Lymphocytes were gated based of SSC and FSC. (B) CD3 negative population was gated from selected lymphocyte population, and gating was determined using isotype controls. (C) NK cell purity was determined based on CD56 positive cells using the CD3 negative population.

**A GANASAPDQLSLALAWNR peptide**

| **Blank**  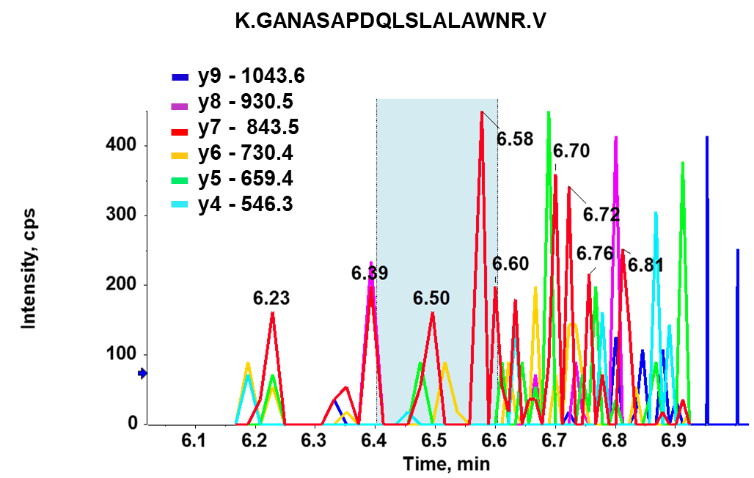 | **Sample 01**  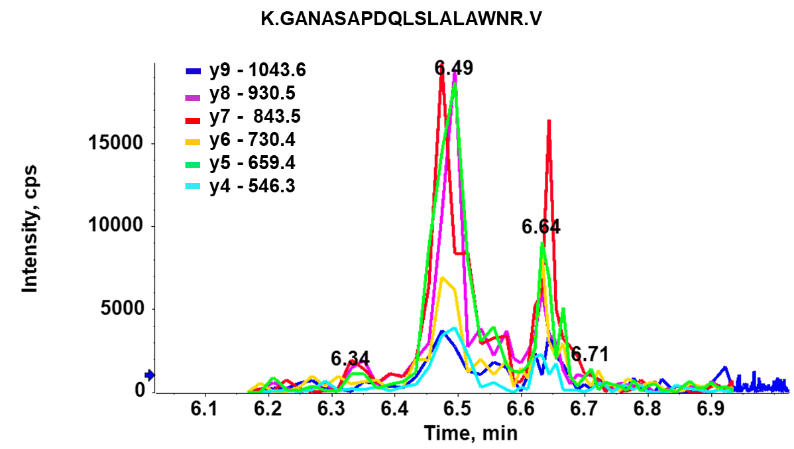 | **Sample 02**  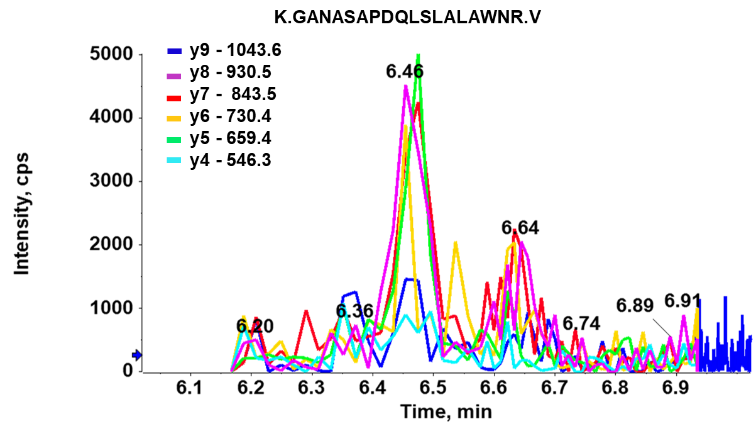 |
| --- | --- | --- |
| **Sample 03**  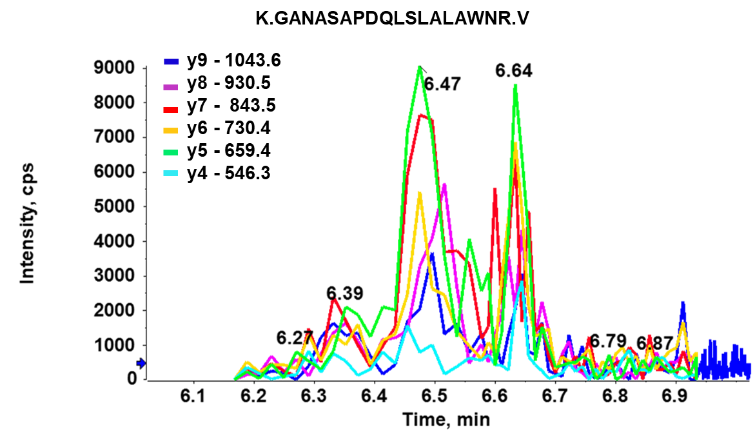 | **Sample 04**  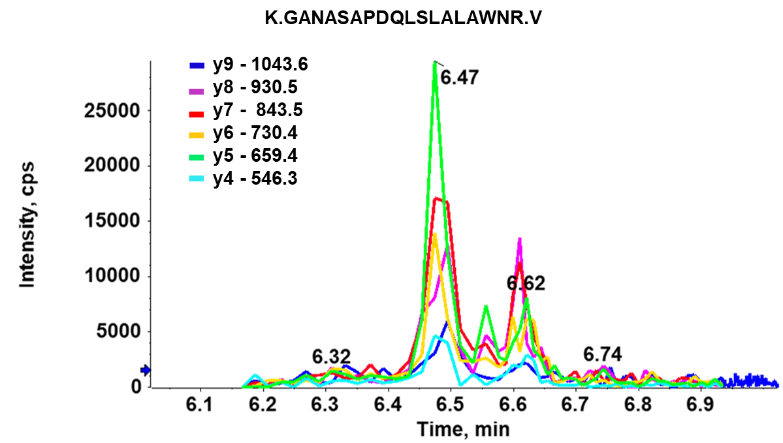 | **Sample 05**  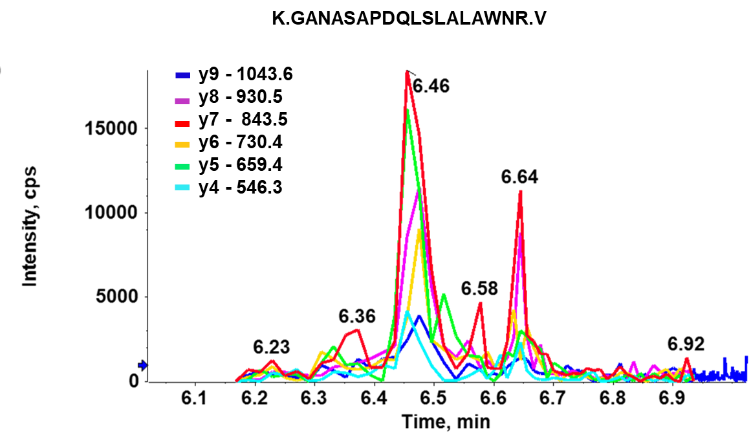 |

| **Sample 06**  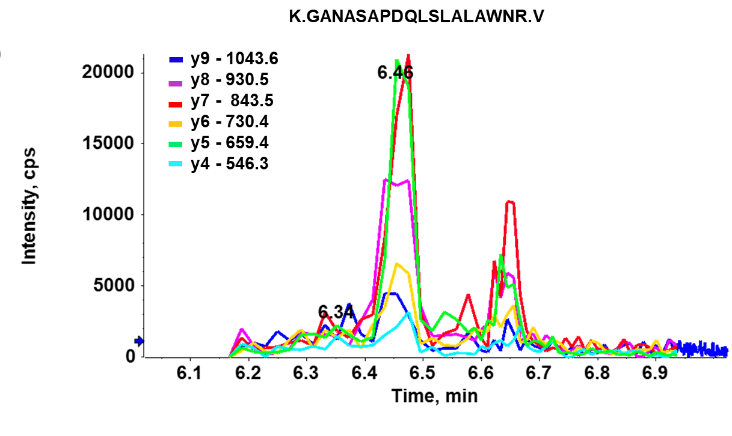 | **Sample 07**  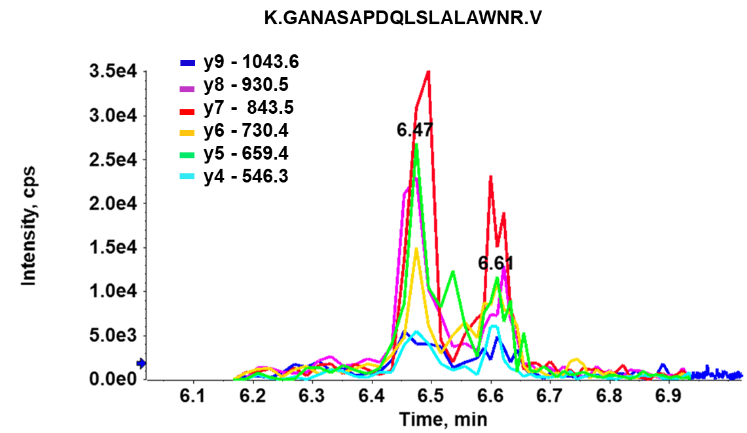 | **Sample 08**  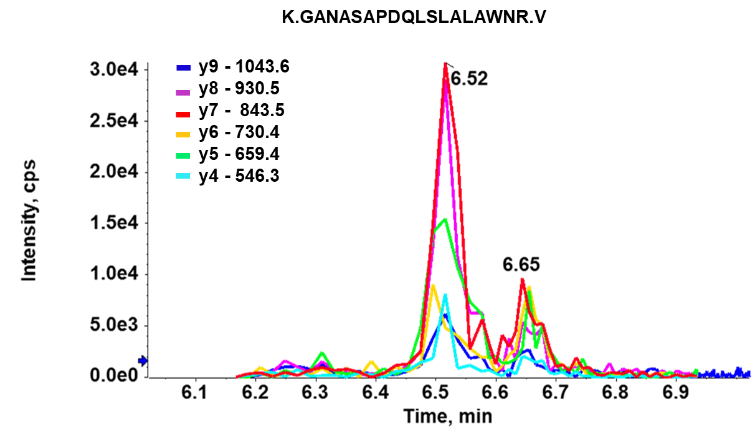 |
| --- | --- | --- |
| **Sample 09**  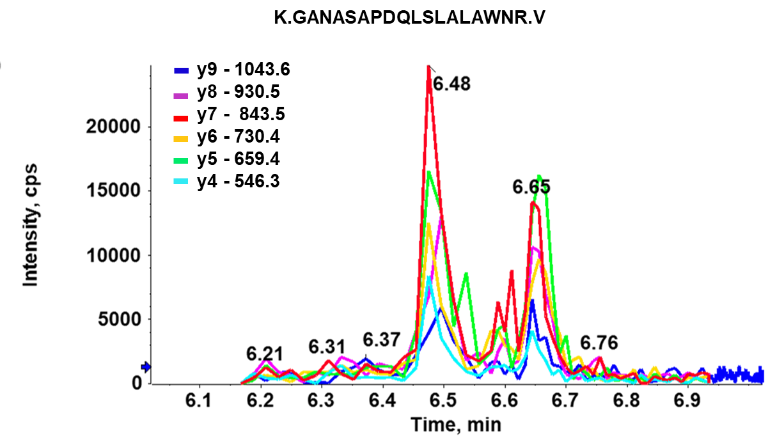 | **Sample 10**  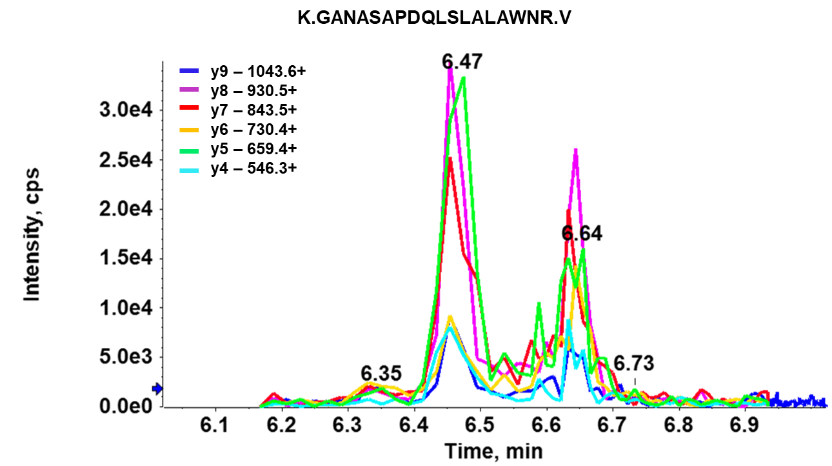 |  |

**B QAILFPNEEPSWK peptide**

| **Blank**  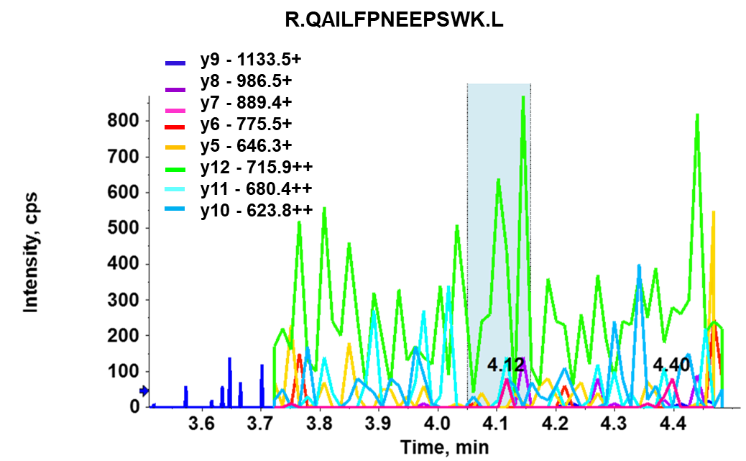 | **Sample 01**  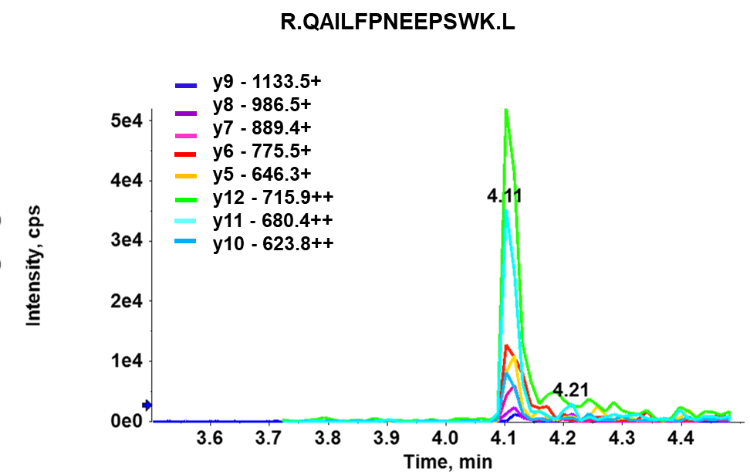 | **Sample 02**  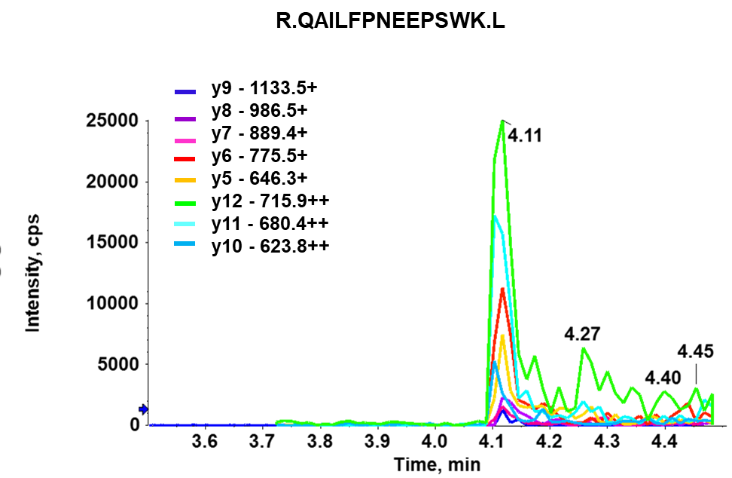 |
| --- | --- | --- |
| **Sample 03**  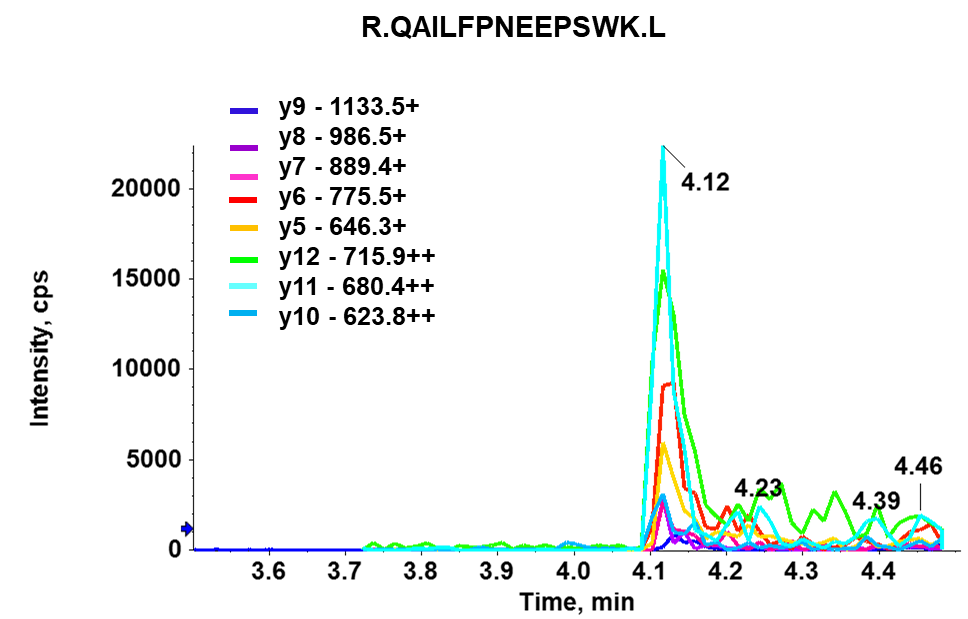 | **Sample 04**  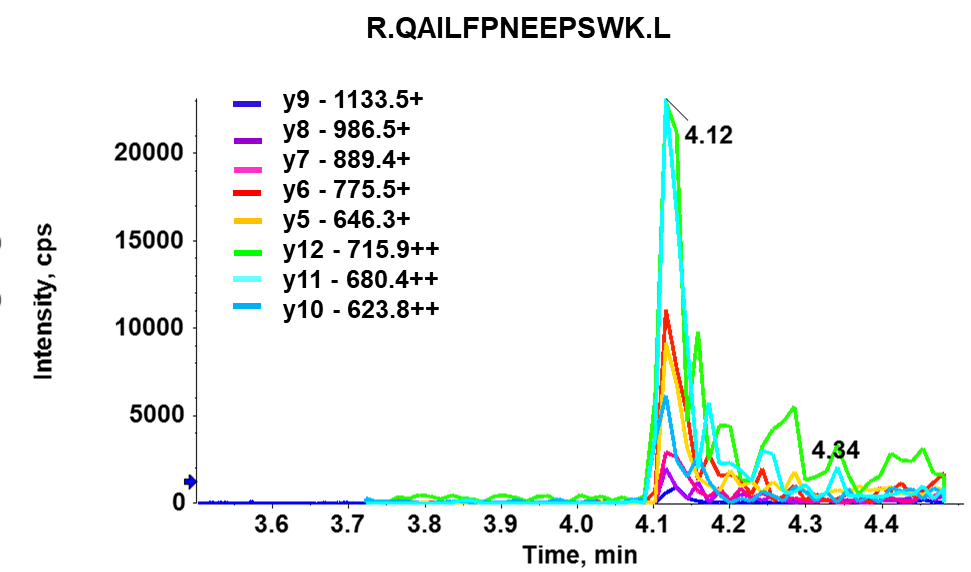 | **Sample 05**  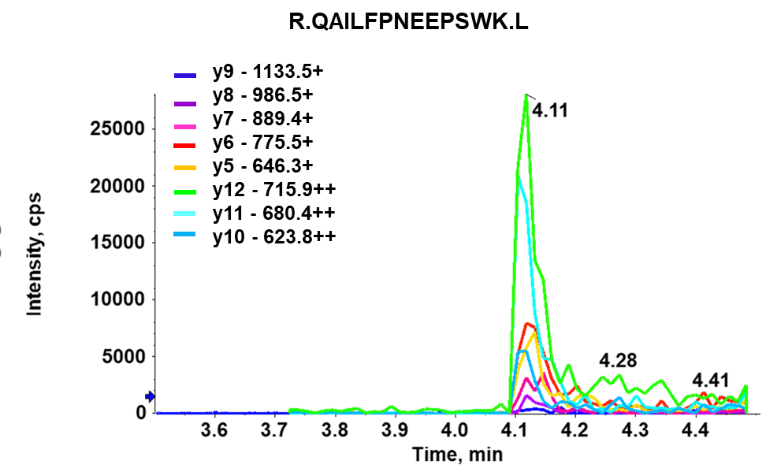 |

| **Sample 06**  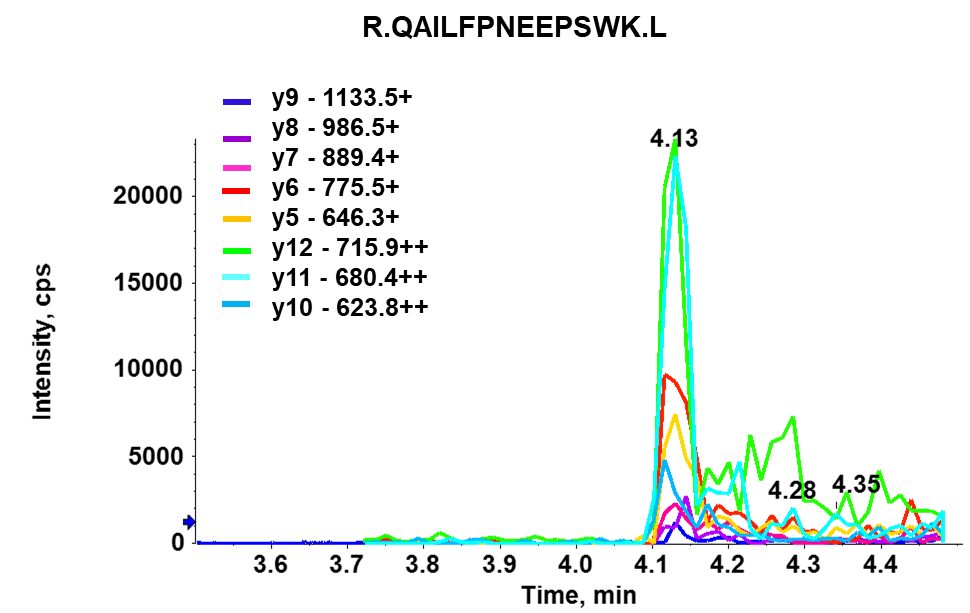 | **Sample 07**  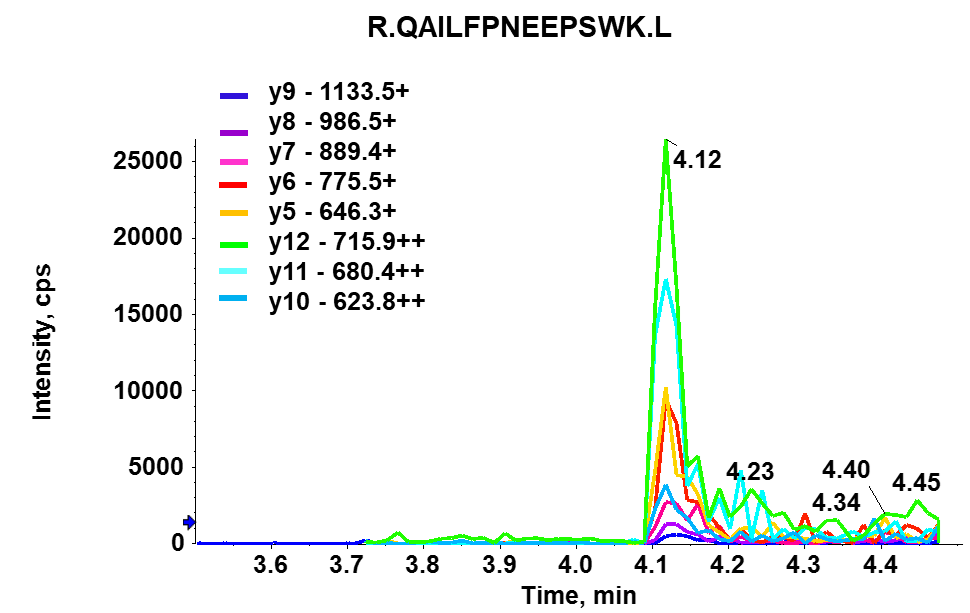 | **Sample 08**  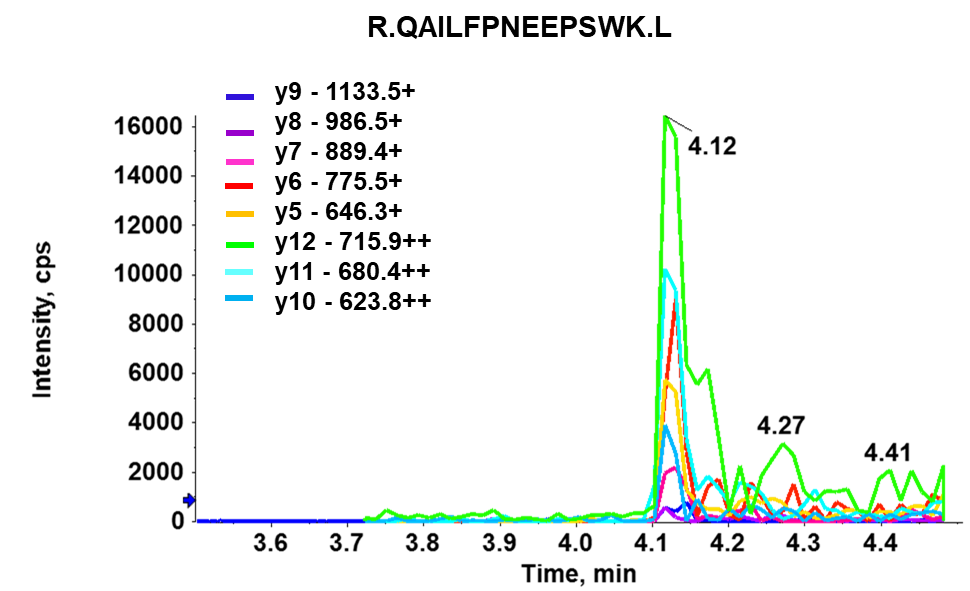 |
| --- | --- | --- |
| **Sample 09**  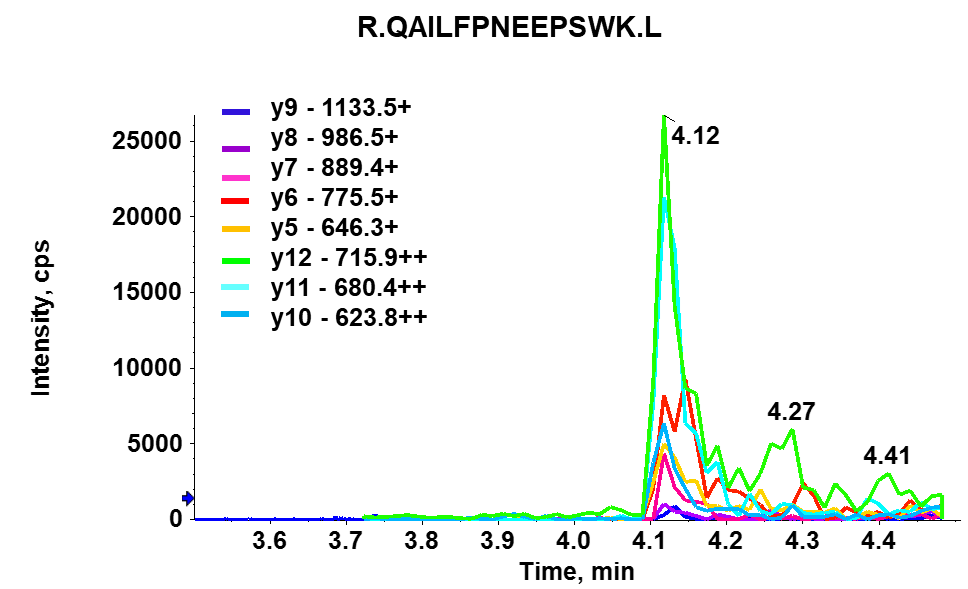 | **Sample 10**  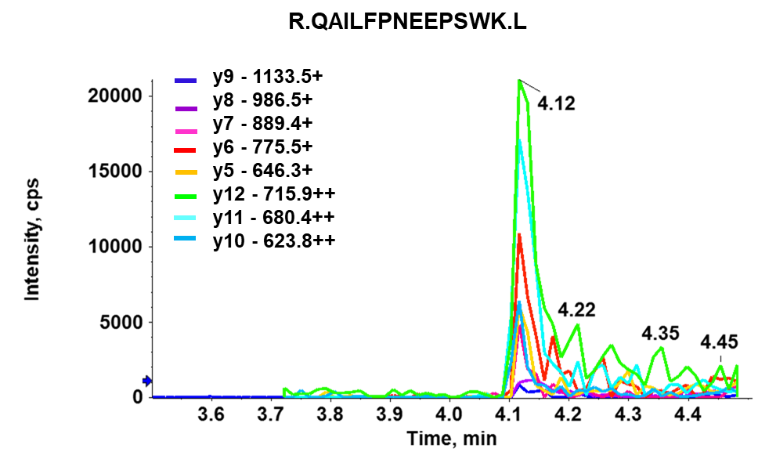 |  |

**C SIDFEDITSMDTR peptide**

| **Blank**  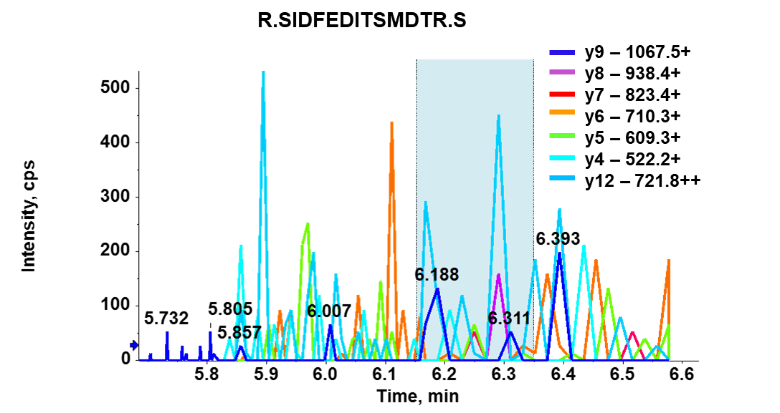 | **Sample 04**  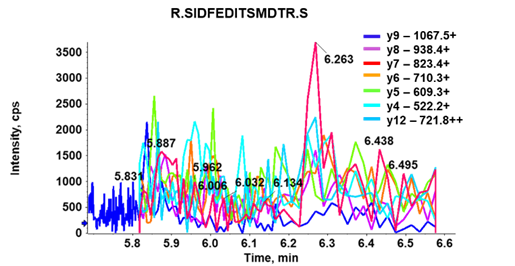 | **Sample 07**  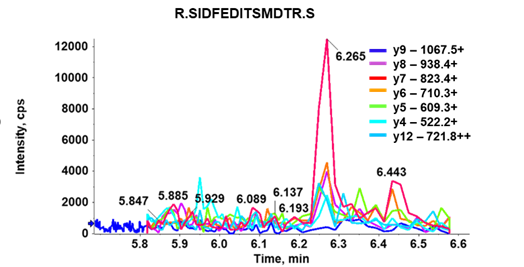 |
| --- | --- | --- |
| **Sample 07**  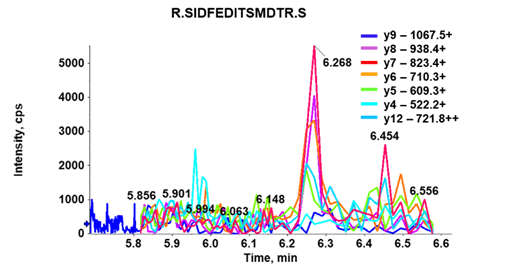 |  |  |

**Figure 2:** Supplementary material 1.1. Detectability of TRPM3 proteotypic peptide sequences across ten samples. All transitions (precursor/ fragment ion pairs) per peptide were monitored over time yielding a set of chromatographic traces with the retention time and relative signal intensities as coordinates. The resulting MRM peaks were then evaluated for their capability to specifically detect target peptides. (A) MRM traces of six transitions for K.GANASAPDQLSLALAWNR.V peptide across 10 samples. (B) MRM traces of eight transitions for R.QAILFPNEEPSWK.L peptide across 10 samples. (C) SIDFEDITSMDTR peptide showed multiple MRM transitions detection at 6.3 minutes in 3 protein samples only
